# Undifferentiated shock in a cirrhotic patient: Ascites matters

**DOI:** 10.1002/ccr3.4880

**Published:** 2021-10-23

**Authors:** Antigoni Xenou, Eugenia Vranou, Konstantinos A. Boulas, Maria Nathanailidou, Eytyxia Kyriakidou, Konstantinos Sitaridis, Isaac Filippidis, Anestis Hatzigeorgiadis

**Affiliations:** ^1^ Department of Radiology General Hospital of Drama Drama Greece; ^2^ Department of General Surgery General Hospital of Drama Drama Greece

**Keywords:** cirrhosis, hemoperitoneum, hepatocellular carcinoma, rupture, shock

## Abstract

In cirrhotic patients with undifferentiated shock, early CT with emphasis in ascitic fluid density should be performed to exclude rare causes of shock such as secondary peritonitis or hemoperitoneum.

## Graphical abstract

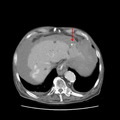

An 86‐year‐old male patient with Child‐Pugh A HBV‐cirrhosis was ambulance transferred with ATLS‐stage 3 shock. Upon admission, the patient was afebrile and had mean arterial pressure (MAP) 50 mmHg, heart rate 77/min, breathing rate 19/min, normal consciousness, Hb 11 g/dl, Na 130 mmol/L, PaO_2_ 86 mmHg, FiO_2_ 29% (nasal cannula 2 L/min), lactic acid 5 mmol/L, and central venous pressure (CVP) 2 cm H_2_O. As no criteria for sepsis and no evidence of upper‐gastrointestinal bleeding were present, the patient considered to have undifferentiated septic or hemorrhagic shock and admitted to the intensive care unit (ICU) with SOFA score 6. Initial resuscitation included administration of lactated Ringer's solution and norepinephrine with target MAP 60 mmHg, albumin 100 g, third‐generation cephalosporin, PPIs, and 2 FFP units with transient response. Whole‐body CT performed as shown in Figure 1.

**FIGURE 1:**
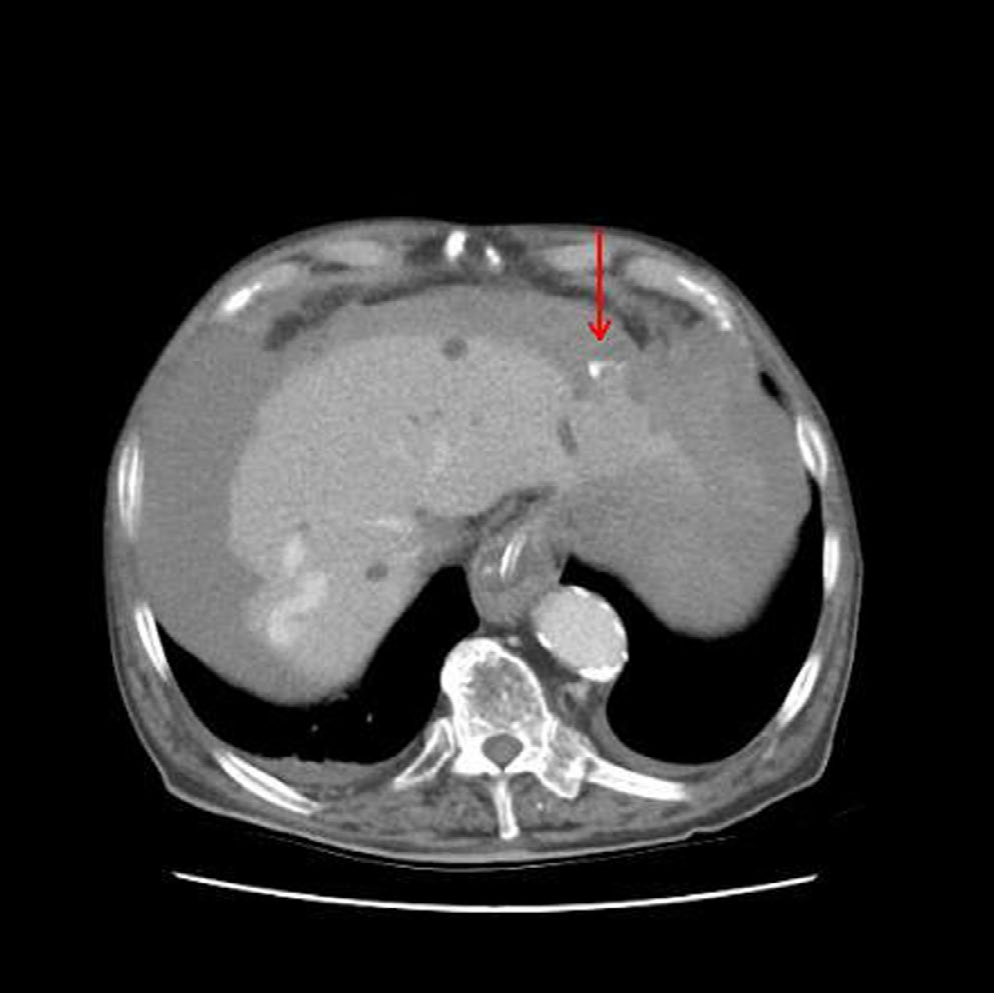
CT revealed no features of pulmonary infection, liver surface nodularity, mild ascites at Morrison and Douglas pouch with 35–45 HU density, a persistent hypoattenuating in all phases peripheral segment III nodule with active intravenous contrast extravasation (red arrow), dilated vena cava, no signs of portal hypertension, no intraperitoneal varices, and no free intraperitoneal air

## 1 QUIZ QUESTION: WHAT IS YOUR DIAGNOSIS?

**FIGURE 2:**
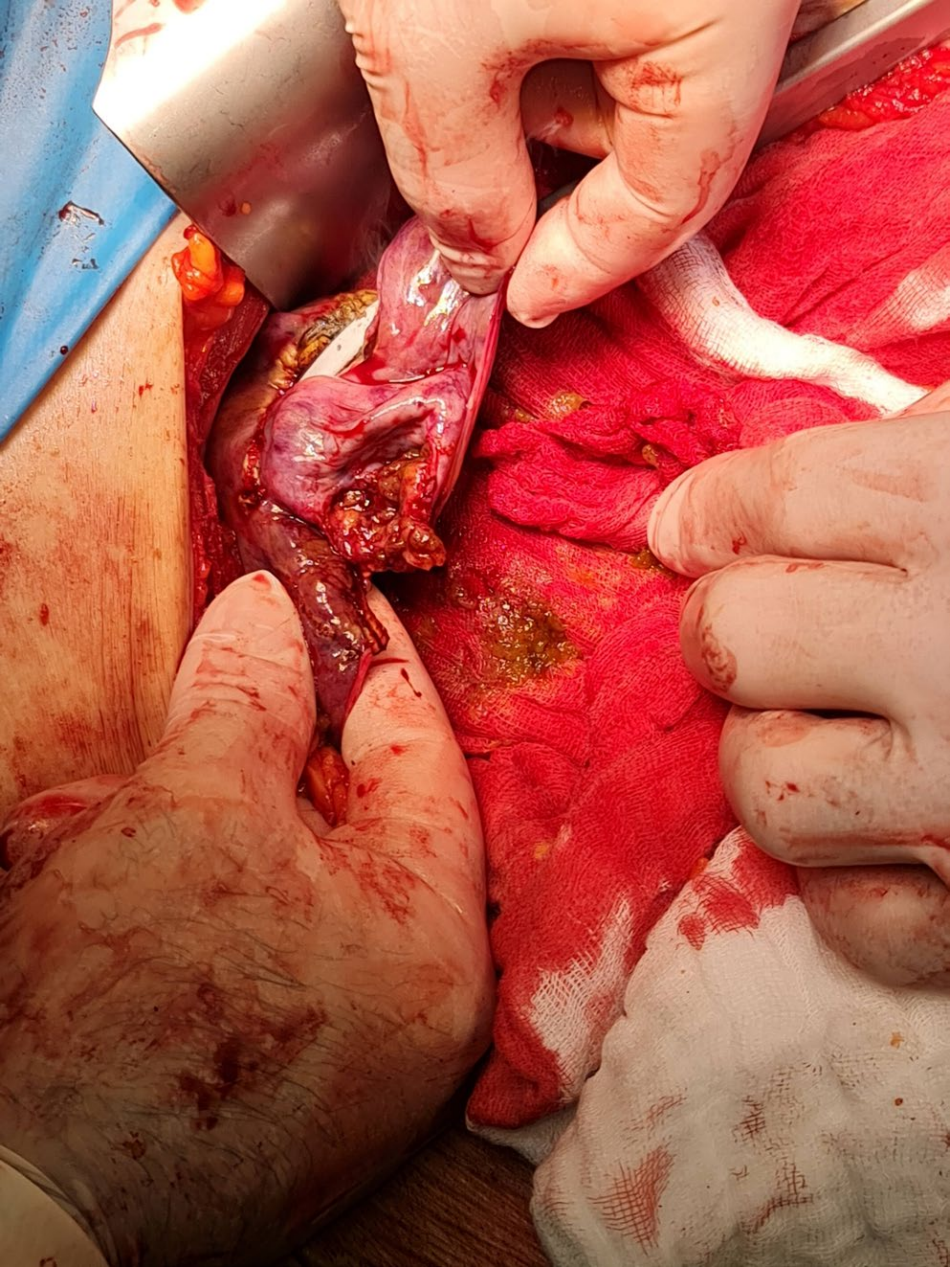
Intraoperative image showing the ruptured peripheral segment III nodule treated with open wedge resection. Biopsy of the surgical specimen revealed an early HCC

Based on the clinical and imaging findings, undifferentiated shock attributed to a ruptured segment III peripheral borderline nodule with hemoperitoneum (Figure 1). As continuous resuscitation resulted in further hemorrhage deterioration due to CVP increase, open wedge resection performed with uneventful recovery (Figure 2). Biopsy of the surgical specimen revealed a ruptured early hepatocellular carcinoma (HCC). Sepsis due to primary peritonitis, pulmonary or urinary tract infection, and portal hypertension‐related upper‐gastrointestinal bleeding are the most common causes of shock and ICU admission in cirrhosis.^1^ In cirrhotic patients with undifferentiated shock and no indications for abdominal paracentesis, early whole‐body CT with emphasis in ascitic fluid density should be performed to exclude rare causes such as secondary peritonitis and hemoperitoneum from a ruptured intraperitoneal varix or HCC.^2^

## CONFLICTS OF INTEREST

The authors declare that they have no conflict of interests.

## AUTHOR CONTRIBUTIONS

All authors equally accessed the data and contributed to the preparation of the manuscript. BKA and HA were equally responsible for making and performing treatment decisions. HA reviewed the manuscript for critical intellectual content and had the final approval.

## STATEMENT OF HUMAN AND ANIMAL RIGHTS

The present article does not contain any studies with human or animal subjects performed by any of the authors.

## CONSENT

Informed consent was obtained from the patient.

## Data Availability

Data sharing is not applicable to this article as no datasets were generated or analyzed during the current study.
